# Cellular Effects of Cyclodextrins: Studies on HeLa Cells

**DOI:** 10.3390/molecules27051589

**Published:** 2022-02-28

**Authors:** Ágnes Rusznyák, Mercédesz Palicskó, Milo Malanga, Éva Fenyvesi, Lajos Szente, Judit Váradi, Ildikó Bácskay, Miklós Vecsernyés, Katalin Szászné Réti-Nagy, Gábor Vasvári, Ádám Haimhoffer, Ferenc Fenyvesi

**Affiliations:** 1 Department of Pharmaceutical Technology, Faculty of Pharmacy, University of Debrecen, Nagyerdei St. 98, H-4032 Debrecen, Hungary; rusznyak.agnes@pharm.unideb.hu (Á.R.); palicsko.mercedesz@gmail.com (M.P.); varadi.judit@pharm.unideb.hu (J.V.); bacskay.ildiko@pharm.unideb.hu (I.B.); vecsernyes.miklos@pharm.unideb.hu (M.V.); retinagykatalin@pharm.unideb.hu (K.S.R.-N.); vasvari.gabor@pharm.unideb.hu (G.V.); haimhoffer.adam@pharm.unideb.hu (Á.H.); 2 Doctoral School of Pharmaceutical Sciences, University of Debrecen, Nagyerdei St. 98, H-4032 Debrecen, Hungary; 3 Institute of Healthcare Industry, University of Debrecen, Egyetem Square 1, H-4032 Debrecen, Hungary; 4 CycloLab Cyclodextrin R&D Laboratory Ltd., Illatos St. 7, H-1097 Budapest, Hungary; malanga@cyclolab.hu (M.M.); fenyvesi.e@cyclolab.hu (É.F.); szente@cyclolab.hu (L.S.)

**Keywords:** HPBCD, RAMEB, endocytosis, lysosomes, autophagy, NF-κB, HeLa

## Abstract

Cyclodextrins are high molecular weight, hydrophilic, cyclic, non-reducing oligosaccharides, applied as excipients for the improvement of the solubility and permeability of insoluble active pharmaceutical ingredients. On the other hand, beta-cyclodextrins are used as cholesterol sequestering agents in life sciences. Recently, we demonstrated the cellular internalization and intracellular effects of cyclodextrins on Caco-2 cells. In this study, we aimed to further investigate the endocytosis of (2-hydroxylpropyl)-beta-(HPBCD) and random methylated-beta-cyclodextrin (RAMEB) to test their cytotoxicity, NF-kappa B pathway induction, autophagy, and lysosome formation on HeLa cells. These derivatives were able to enter the cells; however, major differences were revealed in the inhibition of their endocytosis compared to Caco-2 cells. NF-kappa B p65 translocation was not detected in the cell nuclei after HPBCD or RAMEB pre-treatment and cyclodextrin treatment did not enhance the formation of autophagosomes. These cyclodextrin derivates were partially localized in lysosomes after internalization.

## 1. Introduction

There are numerous applications of cyclodextrins in pharmaceutical technology and drug formulations for the improvement of the water solubility, delivery, and bioavailability of non-soluble drugs with low intestinal permeability [1]. These excipients are increasingly used in the food and cosmetic industries to cover unpleasant flavors, mask taste, and reduce irritation [2,3,4]. On the other hand, beta-cyclodextrins can remove cholesterol from the lipid bilayer of cell membranes [5] causing concentration-dependent cytotoxicity [6] and modulating membrane protein functions such as ABC transporters [7]. HPBCD is taken up by cells through endocytic pathways; it removes the abnormally accumulated cholesterol from Niemann–Pick type C (NPC) mutant cells [8], and it has been a clinically applied drug since 2011 in the treatment of this disease [9]. HPBCD also showed positive effects in Alzheimer’s disease [10], in cardiovascular diseases [11], and it may act as an anticancer agent [12]. These positive effects revealed that cyclodextrins can be considered potential drugs and it is of growing importance to know more about the cellular effects of these cyclodextrin derivatives. HBPCD treatment reduced intracellular cholesterol causing leukemic cell growth inhibition and apoptosis [12], while the antitumor activity of folate-appended methyl-β-cyclodextrin was mediated by the regulation of autophagy [13].

The mechanism of internalization of cyclodextrins was recently characterized both on Caco-2 [14] and HeLa cell lines and it was found that the major routes of internalization are macropinocytosis and clathrin-dependent endocytosis, respectively [15,16,17].

Nuclear factor-κB (NF-κB) is a pleiotropic regulator, which consists of two subunits, p50 and p65 [18]. These subunits are localized in the cytosol in an inactive form, but in the appropriate case, such as inflammatory signals, p65 subunit rapidly translocates into the cell nucleus and regulates many biological processes [19]. The possible stimulation of this major inflammatory pathway caused by the impairing effects of cyclodextrins on cell membrane and cellular components has not been studied yet.

Autophagy plays an important role in the protein degradation of cells by self-digestion. During autophagy, the damaged proteins or the injured cell compartments are removed from the cells via autophagosomes [20,21,22].

This study focused on the investigation of the cellular uptake of HPBCD and RAMEB conjugated with different fluorescent dyes and studied the impact of the internalization of these cyclodextrin derivatives on the cellular functions of HeLa cells. These excipients can affect the functions of the cell membrane as well as proteins such as AMP-activated protein kinase [23], β-amyloid peptid [24], and KV1.3 ion channels by direct interactions [25]. Based on previous results, the purpose of this study was to investigate the effects of short-term (30 min), non-toxic cyclodextrin treatment on cellular functions, which can indicate the acute cellular damage. Another objective of the work was to reveal the differences of cyclodextrin-cellular interactions on different cell types by comparing the results of this study to our previous findings on Caco-2 cells. This is the first study that systematically compares the cellular effects of cyclodextrin derivatives on different cell types and presents the cell type-dependent differences.

## 2. Results

### 2.1. Cytotoxicity

**Figure 1:**
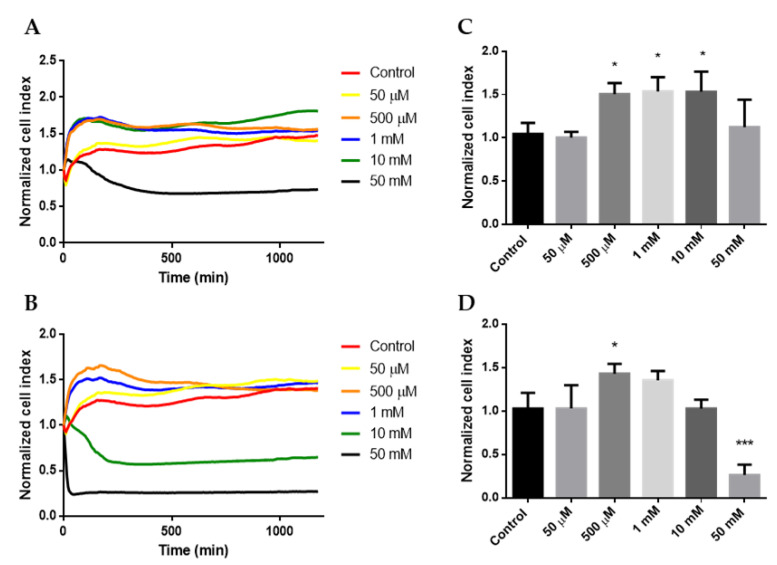
Cytotoxicity of HPBCD (**A**) and RAMEB (**B**) on HeLa cells in the function of time, measured by the RTCA method. The normalized cell index of HPBCD (**C**) and RAMEB (**D**) at 30 min was collected and presented for the applied cyclodextrin concentrations. Mean ± SD is depicted (n = 3). * *p* < 0.05; *** *p* < 0.001.

The RTCA method was used to assess the cytotoxic effects of different cyclodextrin concentrations on HeLa cells. At time t = 0, cyclodextrin solutions were added to the cell layers. Impedance changes were recorded for more than 16 h and expressed as the normalized cell index. A 50 mM HPBCD solution decreased the cell index in the function of time, indicating cytotoxicity (Figure 1A), while RAMEB showed cytotoxic effects at 10 and 50 mM concentrations (Figure 1B). Analyzing the normalized cell index values at 30 min of incubation, we found that treatments with 50 µM HPBCD (Figure 1C) and RAMEB (Figure 1D) did not alter cell viability. Therefore, we used this concentration and incubation time in further experiments.

### 2.2. Investigation of the Cellular Uptake of Cyclodextrins

**Figure 2:**
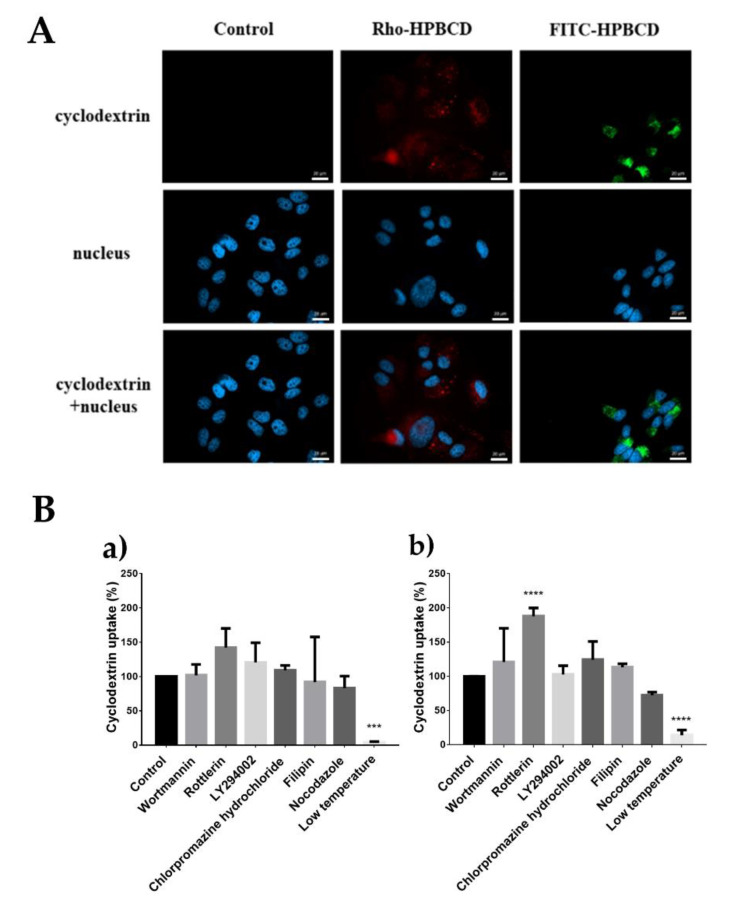
Investigation of the cellular uptake of cyclodextrins. (**A**) FITC-HPBCD and Rho-HPBCD entered HeLa cells and appeared in vesicles in the cytoplasm on fluorescence microscopy images. (Green pixels—FITC-HPBCD, red pixels—Rho-HPBCD, blue pixels—cell nuclei.) Scale bar is 20 µm. (**B**) Flow cytometry analysis of the effect of endocytosis inhibitors and cooling on the internalization of FITC-HPBCD (**a**) and FITC-RAMEB (**b**). Cooling significantly inhibited the internalization process compared to the untreated control. Mean ± SD depicted (n = 3–4), *** *p* < 0.001, **** *p* < 0.0001.

As is shown in Figure 2A, both Rho- and FITC-labelled HPBCD could be detected in the cytosol of HeLa cells and localized in granules after 30 min of incubation. Flow cytometry measurements confirmed the cellular internalization of cyclodextrins and Figure 2B shows that cooling significantly inhibited the endocytosis of these derivatives. Interestingly, rottlerin remarkably increased FITC-RAMEB uptake (Figure 2B(b)), while it only slightly enhanced FITC-HPBCD uptake (Figure 2B(a)). Other inhibitors had no significant effects on the endocytosis of these derivatives.

### 2.3. NF-κB Pathway Activation Study

**Figure 3:**
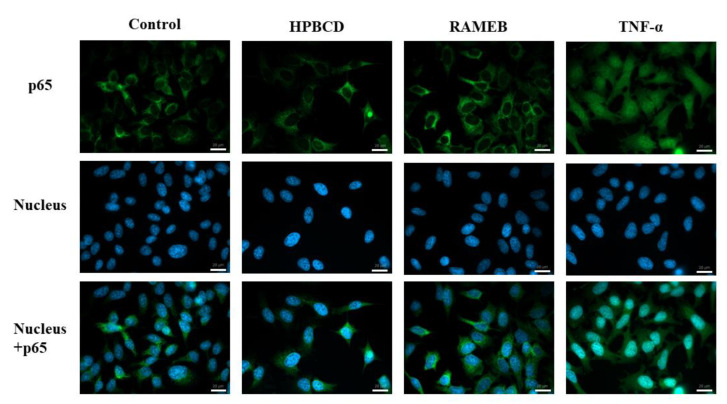
Fluorescence microscopy investigation of the activation of the NF-κB pathway in HeLa cells. The 50 µM cyclodextrin pre-treatment did not stimulate p65 subunit translocation to the cell nuclei, similar to the control sample. TNF-α was a positive control causing p65 appearance in the cell nuclei. (Green pixels—p65 subunit, blue pixels—cell nuclei). Scale bar is 20 µm.

In this experiment, the stimulation of the NF-κB pathway was investigated by immunofluorescence following the translocation of the p65 subunit to the cell nuclei after cyclodextrin or TNF-α treatments. As is shown in Figure 3, the green signal of p65 can be observed only in the cytoplasm after 30 min, non-toxic, 50 µM HPBCD and RAMEB treatments. This stimulus did not induce the translocation of the p65 subunit in HeLa cells; however, the positive control TNF-α treatment caused a strong p65 accumulation in the cell nuclei.

### 2.4. Autophagy Study

**Figure 4:**
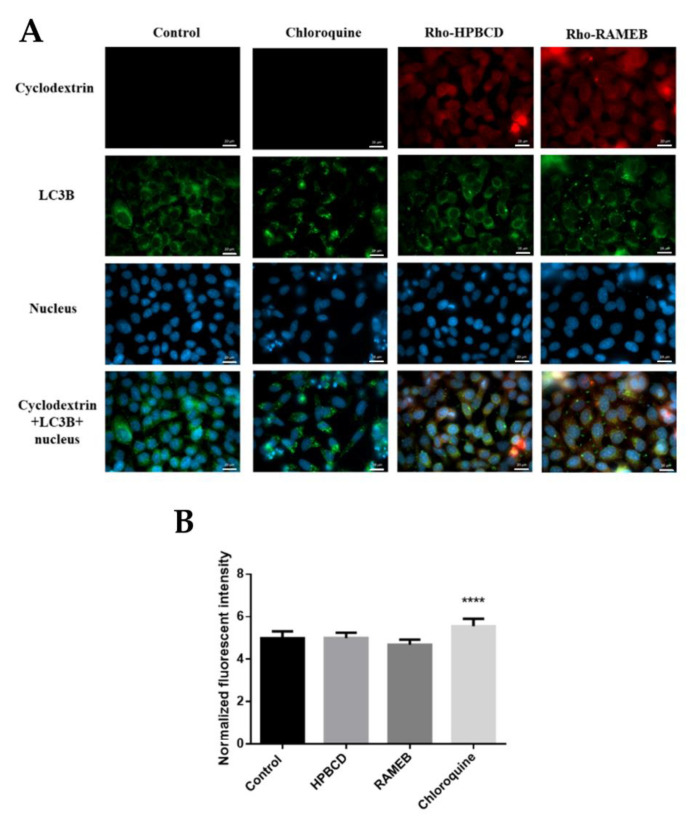
(**A**) Investigation of the autophagosomes in HeLa cells after Rhodamine labelled cyclodextrin or chloroquine treatment, analyzed by fluorescence microscopy. After 50 µM cyclodextrin treatment, the appearance of autophagosomes was similar to the control, while in the case of chloroquine treatment labeling was more intensive. The colocalization of autophagosomes labeled with anti-LC3B antibody and Rho-labeled cyclodextrins appeared with yellow pixels on the merged images. (Green pixels—anti-LC3B antibody, blue pixels—cell nuclei). Scale bar is 20 µm. (**B**) Quantitative analyses of the autophagosomes in HeLa cells after cyclodextrin or chloroquine treatment. Cyclodextrins did not induce the development of autophagosome compared to the control, while chloroquine significantly increased the amount of vesicles. Intensity values were normalized to the cell number and expressed as normalized fluorescence intensity. Results are expressed as mean ± SD, **** *p* < 0.0001.

Figure 4A shows that the pattern of autophagosome staining after rhodamine labelled cyclodextrin treatment was similar to the untreated control; however, after the positive control chloroquine treatment, it was more intensive. Yellow pixels show the colocalization of LC3B (green signal) and Rhodamine labelled HPBCD or RAMEB (red signal) in some vesicles. It reveals the partial localization of cyclodextrins in autophagosomes. The obtained results were confirmed by quantitative measurements. In this experiment, the autophagosome membrane was stained and the fluorescence intensity was measured and normalized to the cell number. As is shown in Figure 4B, HPBCD and RAMEB pre-treatments did not increase, while the chloroquine treatment significantly increased autophagosome formation compared to the control sample.

### 2.5. Investigation of Lysosomes

**Figure 5:**
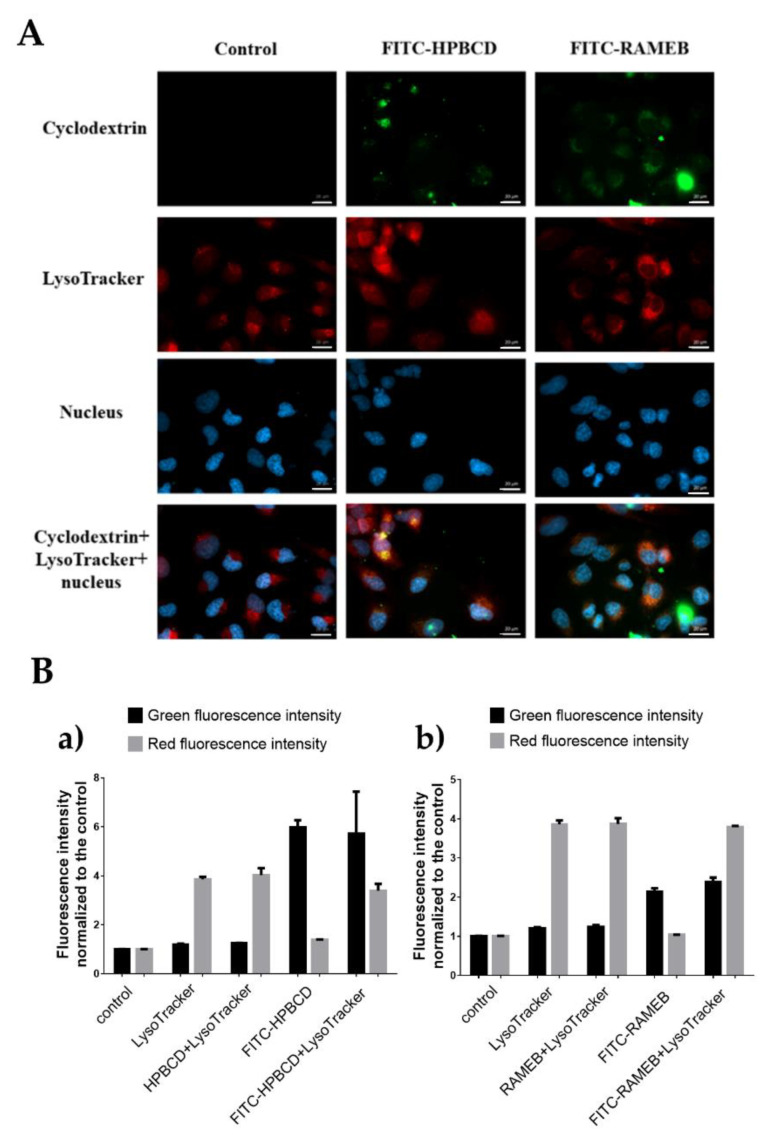
Investigation of lysosomes. (**A**) FITC-labeled HPBCD and RAMEB (green pixels) enter the lysosomes (red pixels) of HeLa cells. Yellow pixels show the cyclodextrin–lysosome colocalization on the fluorescence microscopy images. Scale bar is 20 µm. (**B**) Flow cytometry analysis of lysosomes in HeLa cells after HPBCD (**a**) and RAMEB (**b**) treatments (green fluorescence emitted by the labelled cyclodextrins, while red fluorescence belongs to the LysoTracker^®^). Mean ± SD are depicted, (n = 4).

Figure 5A shows that lysosomes can be detected in HeLa cells by a LysoTracker^®^ and appear as red vesicular staining on the microscopic images. Yellow pixels can be detected in many intracellular vesicles, which shows the colocalization of red (lysosome) and green (FITC-labelled cyclodextrin) signals. Cyclodextrin treatments seemed to stimulate lysosome formation; however, it was not confirmed by the quantitative flow-cytometry analysis. As is shown in Figure 5B, after unlabeled and fluorescein labeled cyclodextrin treatments, the fluorescence intensity of the Lysotracker^®^ was similar to the control sample. After FITC-labelled cyclodextrin treatments, green fluorescence intensity increased, indicating that cyclodextrins entered the cells.

## 3. Discussion

This study reveals important details about the cellular effects of cyclodextrins on HeLa cells, and this is the first systematic comparative study that tests the interactions of cyclodextrins with different cell lines. The data show that there are important differences between the cellular internalization of cyclodextrins (e.g., type of endocytosis, volume of endocytosis) in different cell lines, which can be used in further developments for cell-type specific drug delivery. This work demonstrates the possible selective application of cyclodextrins for endocytosis-mediated cellular uptake. On the other hand, the cellular effects and the intracellular fate of cyclodextrins are poorly studied. More information is needed to reveal the effects of these special macrocycles on cellular processes, especially on the major inflammatory, or metabolic routes, which are connected with endocytosis. To enrich our knowledge in this field, this study focused on the investigation of NF-κB pathway activation and autophagosome and lysosome formation after the endocytosis of cyclodextrins in HeLa cells.

Cyclodextrin treatment has several cellular effects, especially on the cell membrane, causing membrane structure and permeability changes, which at higher concentrations lead to cell death. In RTCA experiments, we found that at certain cyclodextrin concentrations, cell index increased without any further toxicity. A similar phenomenon was detected earlier with RTCA and MTT viability tests [6,26]. In these experiments at non-toxic cyclodextrin concentrations, the cellular uptake of MTT dye and cell index of RTCA measurements exceeded the control value and seemed to cause higher viability or proliferation than in the case of untreated cells. We suppose that the possible explanation is the following: at certain non-toxic cyclodextrin concentrations, the cell membrane is reversibly compromised causing increased membrane permeability or morphological changes, which are reflected in the increased MTT uptake or cell index values. RTCA cell index measurement is based on the attachment of cells on the surface of plate electrodes, and any alterations in cellular morphology, attachment or electrical resistance between cells and plate surface cause cell index changes. The RTCA results presented in Figure 1 seem to support this theory, because there was a non-toxic 50 µM HPBCD concentration, which had no significant effect on cells, as well as non-toxic concentrations, which increased cell index between 500 µM–10 mM, and a toxic, 50 mM HPBCD concentration, which decreased cell index. To avoid any toxic or non-toxic membrane effects on cells, we applied 50 µM cyclodextrin concentration in our further experiments.

Previously, we showed that both Rhodamine and FITC labelled cyclodextrins are internalized by Caco-2 cells via endocytosis [14,16]. In the present study, we applied a non-toxic, 50 µM cyclodextrin concentration for 30 min and showed that Rho and FITC labelled HPBCD entered and localized in granules in the cytoplasm of HeLa cells, similar to Caco-2 cells [14]. The cellular internalization of these derivatives was found to be temperature dependent. On HeLa cells, we could not find the main mechanism of endocytosis, while on Caco-2 intestinal epithelial cells, the main route was the macropinocytosis. On HeLa cells, only the macropinocytosis inhibitor rottlerin [27] has significant effects: it significantly increased the uptake of FITC-RAMEB and slightly enhanced the uptake of FITC-HPBCD. Rottlerin has several cellular effects, and some possible explanations can be proposed. At first, rottlerin in the concentration with 40 min pre-incubation induced autophagy related to apoptosis [28]. Apoptotic cells may take up more cyclodextrin; however, apoptotic cells were excluded from the evaluation by propidium iodide staining. On the other hand, autophagy inducement may cause the formation of autophagosomes, which fuse with lysosomes and may contribute to the cellular accumulation. Finally, inhibition of macropinocytosis with rottlerin may upregulate other endocytotic pathways, such as clathrin-coated uptake, which was reported to be a major pathway in HeLa cells [17]. The other macropinocytosis inhibitors, wortmannin and LY29402 [29], the most commonly used caveola-mediated endocytosis inhibitor filipin [30], and nocodazole, which disrupts the microtubules, had no significant effect on the cellular internalization, similar to Caco-2 cells [14]. Interestingly, according to our measurements by flow cytometry, which measures the whole cell fluorescence intensity, the clathrin-mediated endocytosis inhibitor chlorpromazine [31], had no significant effects on endocytosis. In contrast, using fluorescence microscopy and investigating the morphology, Plazzo, A.P. et al. found that this agent inhibits the endocytic process, and methyl-beta-CD-loaded vesicles remained near the plasma membrane [17].

The activation of the NF-κB pathway by cyclodextrins has not been studied yet. It has only been investigated on LPS stimulated macrophages, where cyclodextrin antagonized the macrophage stimulation [32]. In this study, we found that cyclodextrins in a non-toxic 50 µM concentration did not activate this inflammatory pathway, similarly to Caco-2 cells [14,19]. These results support new information on the safety profiles of cyclodextrins.

Previously, we showed that on Caco-2 intestinal epithelial cells, cyclodextrin pretreatment did not induce autophagy [14]. In this study, we demonstrate that HPBCD and RAMEB pretreatment in a 50 µM concentration did not stimulate the development of autophagosomes, and a non-significant amount of cyclodextrin could be detected in the vesicles of HeLa cells.

On the other hand, similar to our previous results, a significant amount of cyclodextrin was able to enter the lysosomes, but it did not considerably increase the formation of these vesicles. It can be noted that in Caco-2 cells, larger lysosomal vesicles could be identified with higher cyclodextrin content than in HeLa cells.

The main differences between the cellular effects of cyclodextrins on Caco-2 and HeLa cells are summarized in Table 1.

In conclusion, the intracellular compartmentalization of cyclodextrins seems to be similar in Caco-2 and HeLa cells, which suggests that physiological differences of the cell lines are the primary determinants of the internalization and intracellular fate of cyclodextrins. However, some differences between the internalization of HPBCD and RAMEB were detected by the application of endocytosis inhibitors, probably due to the different biological activity of the two derivatives. HPBCD is highly hydrophilic and has less affinity to cholesterol, while RAMEB is considered to be “lipophilic” cyclodextrin with well-known high cholesterol binding affinity. These features can contribute to the altered effects of endocytosis inhibitors.

## 4. Materials and Methods

### 4.1. Materials

The (2-hydroxypropyl)-beta-cyclodextrin (HPBCD) (degree of substitution (DS) ~4.5), random methyl-beta-cyclodextrin (RAMEB) (DS ~12), and the fluorescent derivatives (fluorescein or rhodamine labelled) were from CycloLab Ltd. (Budapest, Hungary). In the case of fluorescently labelled derivatives, the fluorophore (5/6 isomeric mixture) is bound on the primary hydroxyl rim of the cyclodextrin by a chemically stable thioureido group (CycloLab Ltd., Budapest, Hungary).

### 4.2. Cell Culture

The HeLa cell line is a human cervix epithelioid carcinoma cell line (ECACC, Salisbury, UK; passage number 19–40). Cell culture was grown in standardized conditions in Dulbecco’s modified Eagle’s medium (DMEM) containing 10% heat-inactivated fetal-bovine serum, 1% non-essential amino acid, and penicillin-streptomycin solution at 37 °C in an incubator with 5% CO_2_ atmosphere. Cell culture reagents were purchased from Sigma-Aldrich Ltd., Budapest, Hungary.

### 4.3. Investigation of Cytotoxicity

The cytotoxicity of HPBCD and RAMEB solutions was determined by the Real Time Cell Analyser (RTCA) (RTCA DP Instrument), (XCelligence system, ACEA Biosciences Inc., San Diego, CA, USA) as previously demonstrated [14]. Briefly, 5000 cells/well were placed on RTCA plates and after 4 days of growing, cells were incubated with HPBCD and RAMEB solutions in different concentrations. Cell culturing medium was the negative control. Cells were treated with cyclodextrin solutions for 24 h and the cell index was registered every 5 min. Results were expressed as a normalized cell index calculated by the software of the instrument.

### 4.4. Cellular Uptake of Fluorescently Labeled Cyclodextrin Derivatives on HeLa Cells

#### 4.4.1. Fluorescence Microscopy

As previously reported, HeLa cell suspension (50,000 cells/well) was placed on glass cover-slips [14]. When cells reached the appropriate confluence, samples were washed with Hank’s balanced salt solution (HBSS), incubated with 50 µM FITC-HPBCD and Rho-HPBCD (30 min, 37 °C), washed four times, and fixed with a 3.7% paraformaldehyde (PFA) solution for 15 min at room temperature. Then, cells were washed, and cell nuclei were labelled with 283 nM 4′,6-diamidino-2-phenylindole (DAPI) for 10 min at room temperature. At the end of this experiment, the cells were washed again and the cover-slips were mounted to microscope slides. Fluorescence microscopy investigations were conducted with a Zeiss Axioscope A1 (Carl Zeiss AG, Jena, Germany) fluorescence microscope. The following filter sets were used in the microscopy investigations: blue (DAPI): excitation G 365 nm, emission BP 445/50 nm; green (fluorescein): excitation BP 470/40 nm, emission BP525/50 nm; red (rhodamine): excitation BP 546/12 nm, emission BP 575–640 nm.

#### 4.4.2. Flow Cytometry

Endocytosis was confirmed by flow cytometry experiments. These tests were performed as previously described [14]. Briefly, HeLa cells were trypsinized (0.05% trypsin-EDTA), washed with HBSS, and resuspended at a 1 × 10^6^ cells/mL concentration. Cells were pre-treated with or without different endocytosis inhibitors (40 min, 0 °C or 37 °C), then incubated in 50 µM cyclodextrin solutions (30 min, 0 °C or 37 °C) and washed three times with ice-cold HBSS. Then, 1 µg/mL propidium iodide (PI) was added to the samples to stain the dead cells. The fluorescence intensity of the 1 × 10^4^ cells was measured by a Guava Easy Cyte 6HT-2L flow cytometer (Merck Ltd., Darmstadt, Germany). Using green (525/30 nm) and red (695/50 nm) channels, PI positive cells were gated out on a green versus red dot plot and excluded from the evaluation. The mean green fluorescence values of the viable cells were evaluated. The effects of endocytosis inhibitors were compared to the untreated control (100% endocytosis) in three or four independent experiments.

### 4.5. Investigation of the NF-kB Pathway

To examine the stimulation of the NF-κB pathway, the same protocol was used as described previously [14]. Cell suspension (100,000 cells/well) was put on glass cover-slips, and after three days, they were washed with HBSS and incubated with 50 µM HPBCD and RAMEB or 50 ng/mL TNF-α (30 min, 37 °C). After this treatment, samples were rinsed twice with HBSS and fixed with methanol:acetone 1:1 (10 min, room temperature). After fixation, cells were washed with HBSS and the nonspecific binding sites were blocked with Fetal Bovine Serum (FBS) (15 min, 37 °C). After blocking, cells were washed with HBSS and the p65 subunit was labelled with a primary anti-p65 antibody (2 µg/mL, polyclonal rabbit IgG), (1 h, 37 °C). Then, the samples were incubated with the fluorescently labelled secondary antibody (5 µg/mL, Alexa Fluor 488 goat anti-rabbit IgG; 1 h, 37 °C in dark). After both the primary and secondary antibody labelling steps, cells were washed four times with HBSS. At the end of this experiment, cell nuclei were stained with 283 nM DAPI (10 min, 37 °C). The final steps of the sample preparation and the fluorescence microscopy measurements were the same as described in Section 4.4.1. Antibodies used in the experiment were purchased from Sigma-Aldrich Ltd., Budapest, Hungary.

### 4.6. Investigation of Autophagy

#### 4.6.1. Fluorescence Microscopy

In this experiment, autophagy was tested qualitatively by fluorescence microscopy as previously reported [14]. Cells (100,000 cells/well) were grown on glass cover-slips, and after reaching the suitable confluence, they were washed with HBSS and treated with cyclodextrin solutions (50 µM) or a chloroquine solution (100 µM) (positive control) overnight at 37 °C. After treatment, cells were washed, fixed (3.7 % PFA, 15 min, room temperature), and permeabilized (0.2 % Triton-X, 15 min, room temperature). After each step, cells were washed twice with HBSS. Then, the intracellular LC3B molecule was labelled with primary antibody (0.5 µg/mL, rabbit polyclonal antibody against LC3B; LC3B antibody kit for autophagy, Thermo Fisher Scientific, Waltham, MA, USA), (1 h, 37 °C), and fluorescently labelled secondary antibody (5 µg/mL, Alexa Fluor 488 goat anti-rabbit, Sigma-Aldrich Ltd., Budapest, Hungary) (1 h, 37 °C in dark). After labelling, cells were washed with HBSS three times, and the cell nuclei were marked with 283 nM DAPI. The final steps of the sample preparation and the fluorescence microscopy measurements were the same as described in Section 4.4.1.

#### 4.6.2. Microplate Reader

As we described previously [14], autophagy was tested quantitatively by CYTO-ID Autophagy Detection Kit (Enzo Life Sciences, Farmingdale, NY, USA). Cells were grown in a black 96-well plate (10^4^ cell/well). After reaching the suitable confluence, cells were washed with PBS and treated with HPBCD and RAMEB (50 µM) or chloroquine solutions (100 µM, positive control) overnight at 37 °C. After the treatment, cells were washed with PBS and stained according to the instructions of the Kit (1 µL CYTO-ID^®^ Green Detection Reagent and 1 µL Hoechst 33342 Nuclear Stain in 1 mL buffer was added to cells for 30 min at 37 °C). After this step, the samples were washed with PBS and the cellular fluorescence was measured with a FLUOstar Optima microplate reader (BMG Labtech, Offenburg, Germany). Green fluorescence intensity (CYTO-ID^®^ Green Detection Reagent) was measured at 485/520 nm, while blue fluorescence intensity (Hoechst 33342 Nuclear Stain) was detected at 365/445 nm. Green fluorescence intensity values were normalized to the blue fluorescence intensity values according to the experimental protocol of the Kit.

### 4.7. Investigation of the Lysosomes

#### 4.7.1. Fluorescence Microscopy

For the qualitative analyses of lysosomes, cells (40,000 cells/well) were grown on glass cover-slips for two days. After washing with HBSS, cells were treated with fluorescently labeled HPBCD and RAMEB solutions (50 µM, 30 min, 37 °C). Samples were washed three times with HBSS and incubated with the LysoTracker^®^ reagent (50 nM, 30 min, 37 °C). Cells were again washed thoroughly and fixed. The steps from the fixation were the same as described in Section 4.4.1.

#### 4.7.2. Flow Cytometry

For the quantitative investigation of lysosomes [14], FITC conjugated cyclodextrins were utilized. Cells were trypsinized, washed with HBSS, and resuspended at a density of 1 × 10^6^ cells/mL. Cells were pre-incubated with cyclodextrin solutions (50 µM, 30 min, 37 °C) and stained with the LysoTracker^®^ reagent (50 nM, 30 min, 37 °C). Samples were washed with HBSS at 4 °C and fixed with 1% PFA (15 min, room temperature). Single-cell fluorescence analysis was performed by a Guava Easy Cyte 6HT-2L flow cytometer (Merck Ltd., Darmstadt, Germany). The fluorescence of FITC-labeled cyclodextrins was measured as follows: excitation 488 nm, emission 525/30 nm. The LysoTracker ^®^ was detected at 695/50 nm.

### 4.8. Statistical Analysis

For statistical analysis, GraphPad Prism 5 software (GraphPad Software Inc., San Diego, CA, USA) was applied. Means ± SD are presented and ANOVA was used to compare the groups. At *p* < 0.05, differences were considered to be significant.

## Figures and Tables

**Table 1:** Similarities and differences of the cellular effects of cyclodextrins investigated on Caco-2 and HeLa cell lines. Normalized fluorescence intensities were calculated from the flow cytometry experiments, where the fluorescence intensities of the cyclodextrin-treated samples were normalized to the autofluorescence of cells. The applied methods are indicated as abbreviations: FM—fluorescence microscopy; FC—flow cytometry; MR—microplate reader.

	Caco-2 Cell Line	HeLa Cell Line
**Cytotoxicity of cyclodextrins**	50 mM HPBCD and 10 mM RAMEB were cytotoxic (RTCA method)	
**Cellular internalization of fluorescently labeled cyclodextrins**	Both fluorescein and rhodamine labeled HPBCD and RAMEB were localized in vesicles in the cytoplasm. (FM)	
	Normalized fluorescence intensity (FC, 50 µM): FITC-HPBCD: 35.63 ± 2.72 FITC-RAMEB: 4.96 ± 1.23	Normalized fluorescence intensity (FC, 50 µM): FITC-HPBCD: 8.55 ± 2.26 FITC-RAMEB: 2.13 ± 0.33
	The internalization of cyclodextrins was significantly inhibited at 0 °C. (FC)	
	Rottlerin significantly reduced the endocytosis both of FITC-HPBCD and FITC-RAMEB. (FC)	Rottlerin significantly increased the internalization of FITC-RAMEB. (FC)
	Chlorpromazine significantly increased the internalization of the cyclodextrins. (FC)	Chlorpromazine had no significant effect on the cellular uptake of cyclodextrins. (FC)
	Type of cyclodextrin endocytosis: fluid phase endocytosis predominates.	Type of endocytosis: its precise determination requires further experiments, presumably several simultaneous processes including clathrin-dependent endocytosis
**Investigation of lysosomes**	Cyclodextrin tested: Rho-HPBCD, Rho-RAMEB	Cyclodextrins tested: FITC-HPBCD, FITC-RAMEB
	Cyclodextrins entered the lysosomes, did not show more intense staining compared to the control and did not induce the formation of lysosomes. (FM, FC)	
**Investigation of the NF-κB pathway**	p65 translocation was not detected to the nucleus and induction of the NF-κB inflammatory pathway. (FM)	
**Investigation of the autophagy**	Based on qualitative and quantitative studies, neither Rho-HPBCD nor Rho-RAMEB induced the formation of autophagosomes and autophagy. The effect of chloroquine (used as a positive control) was significantly different from that of the control sample. (FM, MR)	

## Data Availability

Not applicable.

## Sample Availability

Samples of the compounds are not available from the authors.

## References

[1] Kurkov S.V., Loftsson T. (2013). Cyclodextrins. Int. J. Pharm..

[2] Szejtli J. (2004). Past, present, and future of cyclodextrin research. Pure Appl. Chem..

[3] Szejtli J. (1997). Utilization of cyclodextrins in industrial products and processes. J. Mater. Chem..

[4] Crini G., Fourmentin S., Fenyvesi É., Torri G., Fourmentin M., Morin-Crini N. (2018). Cyclodextrins, from molecules to applications. Environ. Chem. Lett..

[5] Kilsdonk E.P.C., Yancey P.G., Stoudt G.W., Bangerter F.W., Johnson W.J., Phillips M.C., Rothblat G.H. (1995). Cellular cholesterol efflux mediated by cyclodextrins. J. Biol. Chem..

[6] Kiss T., Fenyvesi F., Bácskay I., Váradi J., Fenyvesi É., Iványi R., Szente L., Tósaki Á., Vecsernyés M. (2010). Evaluation of the cytotoxicity of β-cyclodextrin derivatives: Evidence for the role of cholesterol extraction. Eur. J. Pharm. Sci..

[7] Fenyvesi F., Fenyvesi É., Szente L., Goda K., Bacsó Z., Bácskay I., Váradi J., Kiss T., Molnár É., Janáky T. (2008). P-glycoprotein inhibition by membrane cholesterol modulation. Eur. J. Pharm. Sci..

[8] Rosenbaum A.I., Zhang G., Warren J.D., Maxfield F.R. (2010). Endocytosis of beta-cyclodextrins is responsible for cholesterol reduction in Niemann-Pick type C mutant cells. Proc. Natl. Acad. Sci. USA.

[9] Ory D.S., Ottinger E.A., Farhat N.Y., King K.A., Jiang X., Weissfeld L., Berry-Kravis E., Davidson C.D., Bianconi S., Keener L.A. (2017). Intrathecal 2-hydroxypropyl-β-cyclodextrin decreases neurological disease progression in Niemann-Pick disease, type C1: A non-randomised, open-label, phase 1–2 trial. Lancet.

[10] Vecsernyés M., Fenyvesi F., Bácskay I., Deli M.A., Szente L., Fenyvesi É. (2014). Cyclodextrins, Blood–Brain Barrier, and Treatment of Neurological Diseases. Arch. Med. Res..

[11] Zimmer S., Grebe A., Bakke S.S., Bode N., Halvorsen B., Ulas T., Skjelland M., De Nardo D., Labzin L.I., Kerksiek A. (2016). Cyclodextrin promotes atherosclerosis regression via macrophage reprogramming. Sci. Transl. Med..

[12] Yokoo M., Kubota Y., Motoyama K., Higashi T., Taniyoshi M., Tokumaru H., Nishiyama R., Tabe Y., Mochinaga S., Sato A. (2015). 2-Hydroxypropyl-β-cyclodextrin acts as a novel anticancer agent. PLoS ONE.

[13] Onodera R., Motoyama K., Tanaka N., Ohyama A., Okamatsu A., Higashi T., Kariya R., Okada S., Arima H. (2014). Involvement of autophagy in antitumor activity of folate-appended methyl-β-cyclodextrin. Sci. Rep..

[14] Rusznyák Á., Malanga M., Fenyvesi É., Szente L., Váradi J., Bácskay I., Vecsernyés M., Vasvári G., Haimhoffer Á., Fehér P. (2021). Investigation of the Cellular Effects of Beta-Cyclodextrin Derivatives on Caco-2 Intestinal Epithelial Cells. Pharmaceutics.

[15] Fenyvesi F., Réti-Nagy K., Bacsó Z., Gutay-Tóth Z., Malanga M., Fenyvesi É., Szente L., Váradi J., Ujhelyi Z., Fehér P. (2014). Fluorescently labeled methyl-beta-cyclodextrin enters intestinal epithelial Caco-2 cells by fluid-phase endocytosis. PLoS ONE.

[16] Réti-Nagy K., Malanga M., Fenyvesi É., Szente L., Vámosi G., Váradi J., Bácskay I., Fehér P., Ujhelyi Z., Róka E. (2015). Endocytosis of fluorescent cyclodextrins by intestinal Caco-2 cells and its role in paclitaxel drug delivery. Int. J. Pharm..

[17] Plazzo A.P., Höfer C.T., Jicsinszky L., Fenyvesi É., Szente L., Schiller J., Herrmann A., Müller P. (2012). Uptake of a fluorescent methyl-β-cyclodextrin via clathrin-dependent endocytosis. Chem. Phys. Lipids.

[18] Lawrence T. (2009). The nuclear factor NF-kappaB pathway in inflammation. Cold Spring Harb. Perspect. Biol..

[19] Ueberla K., Lu Y., Chung E., Haseltine W.A. (1993). The NF-kappa B p65 promoter. J. Acquir. Immune Defic. Syndr..

[20] Feng Y., He D., Yao Z., Klionsky D.J. (2014). The machinery of macroautophagy. Cell Res..

[21] Glick D., Barth S., Macleod K.F. (2020). Autophagy: Cellular and molecular mechanisms. J. Pathol..

[22] Mizushima N. (2007). Autophagy: Process and function. Genes Dev..

[23] Dai S., Dulcey A.E., Hu X., Wassif C.A., Porter F.D., Austin C.P., Ory D.S., Marugan J., Zheng W. (2017). Methyl-β-cyclodextrin restores impaired autophagy flux in Niemann-Pick C1-deficient cells through activation of AMPK. Autophagy.

[24] Ren B., Jiang B., Hu R., Zhang M., Chen H., Ma J., Sun Y., Jia L., Zheng J. (2016). HP-β-cyclodextrin as an inhibitor of amyloid-β aggregation and toxicity. Phys. Chem. Chem. Phys..

[25] Kovacs T., Sohajda T., Szente L., Nagy P., Panyi G., Varga Z., Zakany F. (2021). Cyclodextrins Exert a Ligand-like Current Inhibitory Effect on the KV1.3 Ion Channel Independent of Membrane Cholesterol Extraction. Front. Mol. Biosci..

[26] Róka E., Ujhelyi Z., Deli M., Bocsik A., Fenyvesi É., Szente L., Fenyvesi F., Vecsernyés M., Váradi J., Fehér P. (2015). Evaluation of the Cytotoxicity of α-Cyclodextrin Derivatives on the Caco-2 Cell Line and Human Erythrocytes. Molecules.

[27] Sarkar K., Kruhlak M.J., Erlandsen S.L., Shaw S. (2005). Selective inhibition by rottlerin of macropinocytosis in monocyte-derived dendritic cells. Immunology.

[28] Song J., Zhou Y., Gong Y., Liu H., Tang L. (2018). Rottlerin promotes autophagy and apoptosis in gastric cancer cell lines. Mol. Med. Rep..

[29] Falcone S., Cocucci E., Podini P., Kirchhausen T., Clementi E., Meldolesi J. (2006). Macropinocytosis: Regulated coordination of endocytic and exocytic membrane traffic events. J. Cell Sci..

[30] O’Neill M.J., Guo J., Byrne C., Darcy R., O’Driscoll C.M. (2011). Mechanistic studies on the uptake and intracellular trafficking of novel cyclodextrin transfection complexes by intestinal epithelial cells. Int. J. Pharm..

[31] Ivanov A.I. (2008). Pharmacological inhibition of endocytic pathways: Is it specific enough to be useful?. Methods Mol. Biol..

[32] Motoyama K., Arima H., Nishimoto Y., Miyake K., Hirayama F., Uekama K. (2005). Involvement of CD14 in the inhibitory effects of dimethyl-α-cyclodextrin on lipopolysaccharide signaling in macrophages. FEBS Lett..

